# α‐Solanine attenuates chondrocyte pyroptosis to improve osteoarthritis via suppressing NF‐κB pathway

**DOI:** 10.1111/jcmm.18132

**Published:** 2024-02-12

**Authors:** Jinyi Zhou, Jinting Wu, Fangda Fu, Sai Yao, Wenbiao Zheng, Weibin Du, Huan Luo, Hongting Jin, Peijian Tong, Chengliang Wu, Hongfeng Ruan

**Affiliations:** ^1^ Institute of Orthopaedics and Traumatology The First Affiliated Hospital of Zhejiang Chinese Medical University (Zhejiang Provincial Hospital of Traditional Chinese Medicine) Hangzhou China; ^2^ The First People's Hospital of Wenling Taizhou China; ^3^ Xinchang County Hospital of Traditional Chinese Medicine Shaoxing China; ^4^ Department of Orthopedics Taizhou Municipal Hospital Taizhou China; ^5^ Research Institute of Orthopedics The Affiliated JiangNan Hospital of Zhejiang Chinese Medical University Hangzhou China; ^6^ Department of Pharmacy, The Second Affiliated Hospital, School of Medicine Zhejiang University Hangzhou China

**Keywords:** chondrocyte pyroptosis, NF‐κB signalling, nerve ingrowth, osteoarthritis, α‐Solanine

## Abstract

α‐Solanine has been shown to exhibit anti‐inflammatory and anti‐tumour properties; however, its efficacy in treating osteoarthritis (OA) remains ambiguous. The study aimed to evaluate the therapeutic effects of α‐solanine on OA development in a mouse OA model. The OA mice were subjected to varying concentrations of α‐solanine, and various assessments were implemented to assess OA progression. We found that α‐solanine significantly reduced osteophyte formation, subchondral sclerosis and OARSI score. And it decreased proteoglycan loss and calcification in articular cartilage. Specifically, α‐solanine inhibited extracellular matrix degradation by downregulating collagen 10, matrix metalloproteinase 3 and 13, and upregulating collagen 2. Importantly, α‐solanine reversed chondrocyte pyroptosis phenotype in articular cartilage of OA mice by inhibiting the elevated expressions of Caspase‐1, Gsdmd and IL‐1β, while also mitigating aberrant angiogenesis and sensory innervation in subchondral bone. Mechanistically, α‐solanine notably hindered the early stages of OA progression by reducing I‐κB phosphorylation and nuclear translocation of p65, thereby inactivating NF‐κB signalling. Our findings demonstrate the capability of α‐solanine to disrupt chondrocyte pyroptosis and sensory innervation, thereby improving osteoarthritic pathological progress by inhibiting NF‐κB signalling. These results suggest that α‐solanine could serve as a promising therapeutic agent for OA treatment.

## 1 INTRODUCTION

Osteoarthritis (OA) is a prevalent chronic degenerative joint disease that causes pain and disability, affecting millions of patients worldwide and imposing a huge economic burden.^1^ However, the exact aetiology and mechanisms underlying OA remain poorly understood. The existing pharmacologic therapy primarily focuses on pain relief, improving joint function and managing secondary symptoms, while failing to address the complex characteristics of OA. Worse still, long‐term usage of these medications may provide inadequate and unsustainable pain relief and even result in various adverse effects on gastrointestinal, renal and cardiovascular systems.^2^ Consequently, there is an urgent need for preventive and disease‐modifying treatment for OA.

OA is characterized by progressive degeneration of articular cartilage, subchondral bone sclerosis, osteophyte formation, inflammation, aberrant angiogenesis and nerve ingrowth in subchondral bone.^3^, ^4^, ^5^, ^6^ Emerging evidence suggests that pro‐inflammatory cytokines and inflammatory responses contribute to excessive production of catabolic enzymes,^7^ further exacerbating the metabolic imbalance of chondrocytes during OA progression.^8^, ^9^ Extensive works have highlighted the role of IL‐1β in the development of OA and the involvement of nod‐like receptor protein‐3 (NLRP3)‐mediated inflammasome in promoting various joint compartments has garnered significant attention. Both in vitro and in vivo OA model experiments conducted by our team and other researchers have consistently demonstrated that aberrant activation of NF‐κB signalling leads to crucial activation of caspase‐1‐mediated pyroptosis, which drives chondrocyte pyroptosis in OA pathogenesis.^10^, ^11^, ^12^, ^13^, ^14^ Moreover, subchondral bone and articular cartilage act together not only mechanically but biologically to accelerate OA progression.^15^ Emerging evidence reveals that subchondral bone lesions, including angiogenesis and sensory neuron ingrowth, are responsible for cartilage sabotage and pain.^16^, ^17^ Further analysis from clinical and animal model studies has shown that netrin‐1 secreted by osteoclasts induces the growth of calcitonin gene‐related peptide‐positive (Cgrp^+^) sensory nerve during subchondral bone remodelling, serving as a key source of OA‐related pain.^16^, ^17^ Therefore, targeting the inhibition of inflammation response induced by chondrocyte pyroptosis and sensory innervation in subchondral bone might offer potential benefits for pain relief and slowing OA progression.

α‐Solanine is a major glycoalkaloid in nightshade family plants, particularly, in potatoes (*Solanum tuberosum* L.), and has commonly been used in Traditional Chinese Medicine to relieve patients from inflammatory and degenerative disorders due to its desirable properties.^18^ A previous study has demonstrated rational potato administration, such as boiled potato peeling water, could effectively relieve pain and swelling to improve arthritis by inhibiting pro‐inflammatory cytokines expression.^19^ In line with these findings, α‐solanine exhibits various biological effects, including anti‐inflammatory,^20^, ^21^, ^22^ anti‐pulmonary arterial hypertension,^23^ anti‐fungal^24^ and anti‐tumour activities.^25^, ^26^, ^27^ Notably, α‐solanine can inhibit pro‐inflammatory mediators, including IL‐1β, by inactivating NF‐κB signalling, thereby exerting an anti‐inflammatory effect in lipopolysaccharide (LPS)‐induced inflammation of macrophages and animal models.^20^, ^21^, ^22^ It also inhibits matrix metalloproteinases (Mmps) activities by blocking p65 nuclear translocation, thus impeding the migration and invasion of human melanoma cells.^28^ Given these characteristics, α‐solanine present in potatoes holds the potential as an active ingredient for inhibiting OA progression.

The objective of this study is to investigate the direct role of α‐solanine on OA progression. OA model mice that underwent destabilization of the medial meniscus (DMM) surgery were treated with α‐solanine for 8 and 12 weeks. Our findings demonstrate that α‐solanine inhibits subchondral sclerosis and articular cartilage degeneration in OA mice, disrupts chondrocyte pyroptosis in articular cartilage and mitigates aberrant angiogenesis and sensory neuron innervation in subchondral bone, thereby ameliorating the progression of OA. Moreover, α‐solanine inhibits activated NF‐κB signalling in the early stages of OA development, suggesting its potential as a therapeutic agent for OA treatment.

## 2 MATERIALS AND METHODS

### 2.1 Chemicals and reagents

α‐Solanine (93.2% purity) was purchased from ChromaDex Corp (Irvine, CA, USA) and was authenticated by the authors. A voucher specimen (No.: CD20200607) has been deposited at the First Affiliated Hospital of Zhejiang Chinese Medical University (Hangzhou, China). Primary antibodies against Type II collagen (Col2) and 10 (Col10), Mmp3, Mmp13, Endomucin (Emcn), CD31, caspase‐1 and IL‐1β were obtained from Ruiying Biological (Jiangsu, China). Primary antibodies against Cgrp, Netrin and Gasdermin D (Gsdmd) were from Abcam Company Ltd. (Cambridge, MA, USA). Primary antibodies against p65 and phospho‐I‐κBα (Ser32) (p‐I‐κB) were provided by Cell Signalling Technology (Beverly, MA, USA). Unless otherwise specified, all other reagents were supplied by Sigma‐Aldrich (St. Louis, MO).

### 2.2 Animal and OA model

Eight‐week‐old male C57BL/6 mice (*n* = 75, 20 ± 2 g) were purchased from Zhejiang Chinese Medical University laboratory animal research centre (Grade SPF, SCXK (Shanghai): 2017‐0005), kept in a controlled environment with 12 h light/dark cycle and provided ad libitum access to water and chow. All protocols of mouse procedures were approved by the Ethics Committee of Zhejiang Chinese Medical University (NO. ZSLL‐2018‐012).

DMM surgery‐induced OA model was carried out as we previously described.^10^, ^11^ Briefly, the right knee joint's capsule was incised after anesthetization with 1% pentobarbital sodium. The medial meniscotibial ligament was exposed, transected and the medial meniscus was destabilized using microsurgical scissors. The lateral meniscotibial ligament was identified and preserved during the surgery. In control mice, a similar operation was performed on the right knee joint without severing the medial meniscotibial ligament.

### 2.3 Experimental animal design

In Experiment 1, a total of 40 mice were randomly assigned into four groups: Ctrl group (*n* = 10), DMM group (*n* = 10), low‐dose α‐solanine group (*n* = 10) and high‐dose α‐solanine group (*n* = 10). Ten days following the DMM surgery, articular injection of α‐solanine was administered into mice's joint cavity in the low‐dose α‐solanine group (0.5 μmol/L, 6 μL, twice a week) and high‐dose α‐solanine group (2 μmol/L, 6 μL, twice a week), while mice in the Ctrl group and DMM group received an equivalent volume of normal saline. Mice (*n* = 5 per group) were euthanized at 8 or 12 weeks post‐DMM surgery, and the corresponding right knee joints were harvested for further analysis.

In Experiment 2, to determine NF‐κB signalling alterations in the early stage of OA progression, mice (*n* = 5 per time point) that received DMM surgery were sacrificed at 0, 1, 2 and 4 weeks following DMM modelling, and knee joint tissues were harvested for measuring the expression changes of p65 and p‐I‐κB.

In Experiment 3, To assess the effect of α‐solanine on NF‐κB signalling changes in the early stage of OA progression, DMM surgery‐treated mice were treated with different concentrations of α‐solanine (0, 0.5 and 2 μmol/L) for 4 weeks (*n* = 5 in each group), and knee joints were harvested for determining the expression changes of p65 and p‐I‐κB.

### 2.4 Micro‐computed tomography (micro‐CT) analysis

The radiographic changes of knee joints were analysed using Skyscan 1176 Micro‐CT equipment (50 kV, 500 μA) supplied by Bruker Corporation (Kontich, Belgium). The samples were scanned at a resolution of 9 μm, reconstructed using NRecon v1.6, and analysed with CTAn v1.9. A visualization software, CT Vol v2.2, was utilized for visualizing 3D images, and the area between the tibial plateau and proximal tibia growth plate was identified as the region of interest.

### 2.5 Histopathological evaluation

Following Micro‐CT evaluation, all knee joints were fixed in 10% formalin for 24 h, decalcified with 10% ethylene diamine tetraacetate (EDTA) solution (pH 7.4) for 14 days, embedded in paraffin and sliced into 4 μm continuous sections. Safranin O and Fast Green (SO/FG) or haematoxylin & eosin (H&E) staining were performed for histopathological and morphometric analysis. Histologic scoring of OA progression has been performed on Safranin O/Fast Green stained tissue sections according to the Osteoarthritis Research Society International (OARSI)‐recommended scoring system reported by Glasson.^29^ The scores were obtained on a 0–12 scale by multiplying the index of grade and stage (only the tibial plateau is counted). Furthermore, the thickness of the hyaline cartilage (HC) and calcified cartilage (CC) region of the tibia were measured on H&E‐stained sections. The thickness is equal to the total area divided by the width. All analyses were performed in a blinded manner by a previously trained investigator.

### 2.6 Immunohistochemistry (IHC) and immunofluorescence (IF) analyses

The IHC analysis was performed using SP Link Detection Kits (ZSGB‐BIO, China) following the manufacturer's instructions. Briefly, sections were deparaffinized and rehydrated, and subjected to antigen retrieval with 10 mM sodium citrate at 60°C for 15 min. Then, endogenous peroxidase activity was quenched with 3% H_2_O_2_ for 10 min and sequential incubation with normal goat serum was conducted for 30 min. Then sections were incubated with primary antibody, including Col2 (1/200), Mmp3 (1/400), Mmp13 (1/400) and Col10 (1/1000) at 4°C overnight, followed by incubation with biotinylated secondary antibody and horseradish peroxidase‐conjugated streptavidin‐biotin staining. Immunoreactivity was visualized with diaminobenzidine (DAB) and counterstain with haematoxylin. For IF analysis, primary antibodies against Emcn (1/200), CD31 (1/200), p65 (1/200) and p‐I‐κB (1/200) were incubated at 4°C overnight, followed by incubation with fluorescence‐conjugated secondary antibodies (Sungene Biotech, Tianjin, China). After counterstaining with DAPI, the sections were imaged under a Carl Zeiss fluorescence microscope (Göttingen, Germany). Each experiment was repeated in triplicates. Quantitative histomorphometric analysis was conducted in a blinded manner using Image‐Pro Plus Software version 6.0 (Media Cybernetics Inc, Rockville, MD, USA) as we previously described.^10^, ^11^, ^30^

### 2.7 Statistical analyses

Data were presented as the mean ± SD and analysed using one‐way analysis of variance (ANOVA) followed by Tukey's multiple comparisons test. All analyses were performed using GraphPad Prism 8 software (GraphPad Software Inc., San Diego, CA, USA). *p* < 0.05 was considered statistically significant.

## 3 RESULTS

### 3.1 α‐solanine ameliorates OA progression induced by DMM surgery

**FIGURE 1:**
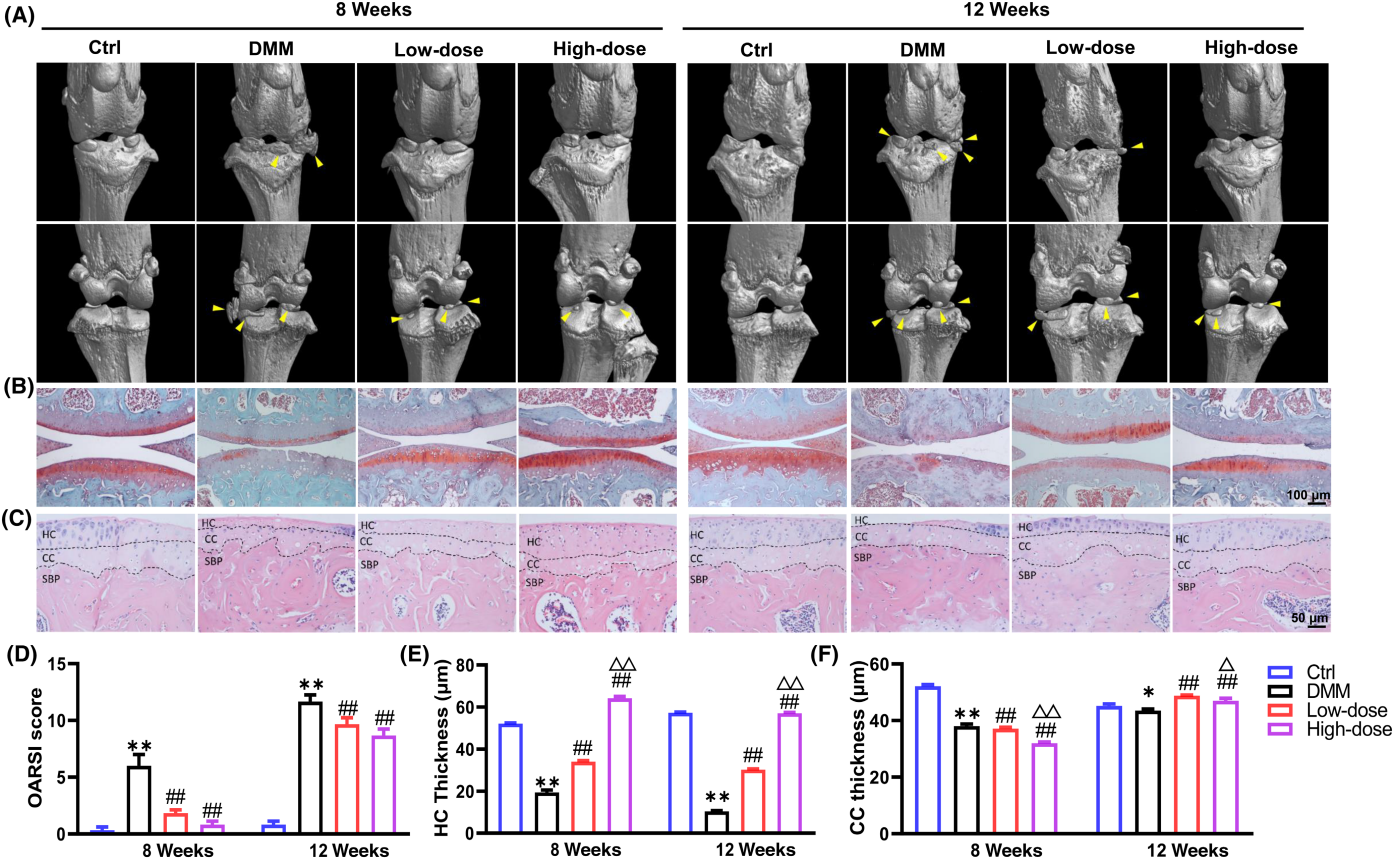
α‐Solanine mitigates DMM surgery‐induced mouse OA progression. (A) Representative three‐dimensional (3D) reconstruction images of anterior and posterior coronal views of subchondral bone in the tibia at 8 and 12 weeks post‐OA modelling. Yellow arrows indicate periarticular osteophytes. (B, C) Safranin O/Fast green and H&E staining results of arthritic cartilage. (D) OARSI score of articular cartilage in B. (E, F) The thickness measurements of HC and CC in tibial cartilage. DMM‐operated mice were treated with low‐dose (0.5 μM) and high‐dose (2 μM) α‐solanine, respectively. *n* = 5 per group. Data are presented as mean ± SD. *p < 0.05, **p < 0.01, compared with Ctrl mice; ^##^ *p* < 0.01, ^##^ *p* < 0.01, compared with OA mice; ^△^ *p* < 0.05, ^△△^ *p* < 0.001, compared with mice receiving low‐dose α‐Solanine (0.5 μM).

To assess the protective effects of α‐solanine against OA progression, two concentrations of α‐solanine (0.5 and 2 μmol/L) were intra‐articularly administered to mice 10 days post‐DMM surgery. The radiographic changes in OA mice were initially evaluated using micro‐CT analysis. We found that OA mice exhibited rough joint surface and osteophyte formation at 8 and 12 weeks after DMM surgery, whereas α‐solanine administration effectively reversed these alterations in a dose‐dependent manner (Figure 1A). Meanwhile, histopathological and morphometric analyses of knee joints were performed using SO/FG and H&E staining. The results of SO/FG staining demonstrated that α‐solanine significantly reduced superficial cartilage damage, erosion, substantial proteoglycan loss and apparent hypocellularity in OA mice (Figure 1B). Furthermore, DMM surgery‐treated mice exhibited higher OARSI scores than Ctrl mice, whereas α‐solanine significantly ameliorated these changes. Unexpectedly, mice treated with α‐solanine for 12 weeks displayed higher OARSI scores than those treated for 8 weeks (Figure 1D). In addition, H&E staining results showed that α‐solanine, particularly at a high dose, effectively reversed the reduction of HC in articular cartilage of OA mice (Figure 1C,E,F).

### 3.2 α‐Solanine prevents matrix degradation in the articular cartilage in OA mice

**FIGURE 2:**
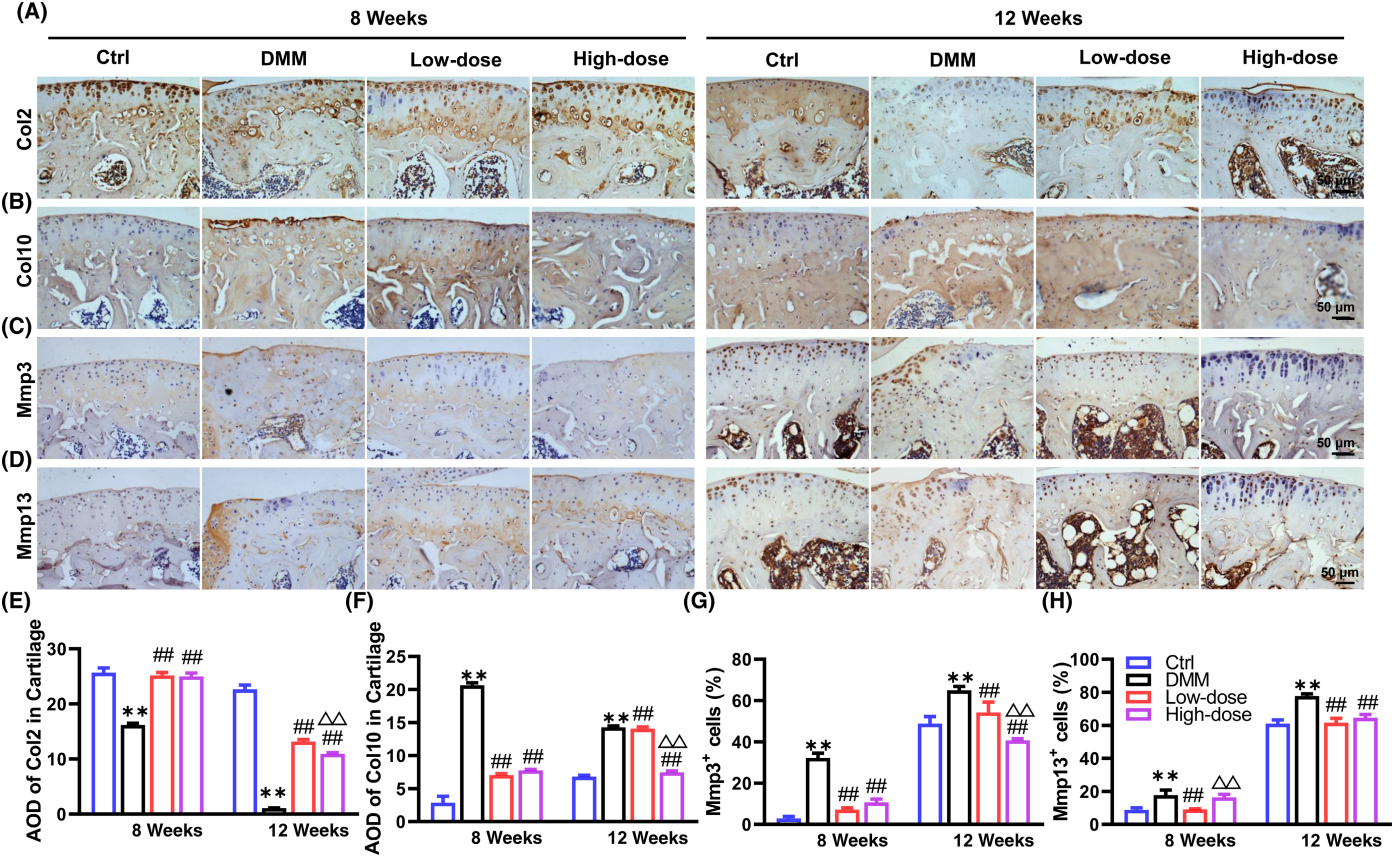
α‐Solanine attenuates cartilage degradation in OA mice. (A–H) IHC staining and quantitative results of Col2, Col10, Mmp3 and Mmp13 in articular cartilage at tibial plateau 8‐ and 12‐week post‐DMM surgery. Data are presented as mean ± SD. Three independent experiments were performed in triplicates. *n* = 5 per group. **p < 0.01, compared with Ctrl mice; ^##^ *p* < 0.01, compared with OA mice; ^△△^ *p* < 0.01, compared with mice receiving low‐dose α‐solanine (0.5 μM).

To identify the influence of α‐solanine on cartilage matrix metabolism of OA mice, we determined the expression of Col2, Col10, Mmp3 and Mmp13 using IHC analysis. The corresponding results showed that α‐solanine reversed the decreased Col2 and increased Col10, Mmp3 and Mmp13 expression in the articular cartilage of OA mice (Figure 2A–D), indicating that α‐solanine could improve the metabolic homeostasis in the articular cartilage of OA mice. Of note, a 12‐week treatment with α‐solanine exhibited less protection against matrix degradation in articular cartilage compared to an 8‐week treatment (Figure 2E–H).

### 3.3 α‐solanine attenuates chondrocyte pyroptosis in OA mice

**FIGURE 3:**
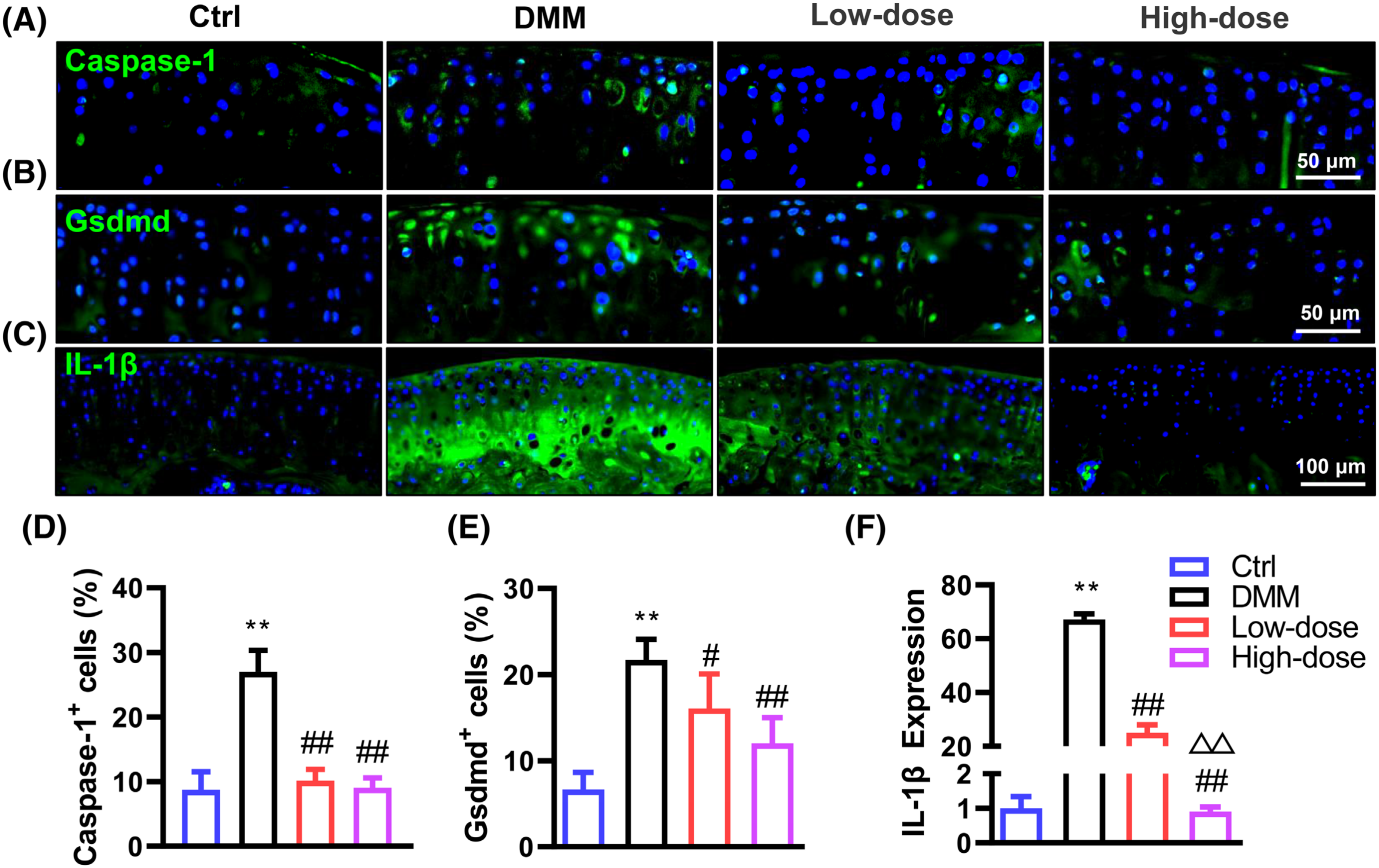
α‐Solanine reduces aberrant chondrocyte pyroptosis in articular cartilage. (A–F) IF staining and quantitative results of caspase‐1, Gsdmd and IL‐1β in articular cartilage at the tibial plateau at 12 weeks post‐DMM surgery. Data are presented as mean ± SD. Three independent experiments were performed in triplicates. *n* = 5 per group. **p < 0.01, compared with Ctrl mice; ^#^ *p* < 0.05, ^##^ *p* < 0.01, compared with OA mice; ^△△^ *p* < 0.001, compared with mice receiving low‐dose α‐solanine (0.5 μM).

Our recent studies have demonstrated that activated chondrocyte pyroptosis is involved in the progression of OA.^10^, ^11^, ^30^ To evaluate whether α‐solanine can inhibit chondrocyte pyroptosis in OA mice, we determined key proteins involved in caspase‐1‐mediated inflammasome signalling (caspase‐1, Gsdmd and IL‐1β) using IF analysis. Consistent with our previous results, increased expressions of Caspase‐1, Gsdmd and IL‐1β were observed in articular chondrocytes of OA mice, whereas α‐solanine significantly reduced Caspase‐1, Gsdmd and IL‐1β in a dose‐dependent manner (Figure 3A–F). Notably, treatment with high or low doses of α‐solanine reduced caspase‐1 levels in the articular cartilage of OA mice to levels comparable to those in Ctrl mice. These data suggest that the improvement of cartilage matrix content in α‐solanine‐treated OA mice may partly result from the suppression of chondrocyte pyroptosis.

### 3.4 α‐Solanine suppresses angiogenesis and sensory nerve ingrowth in subchondral bone of OA mice

**FIGURE 4:**
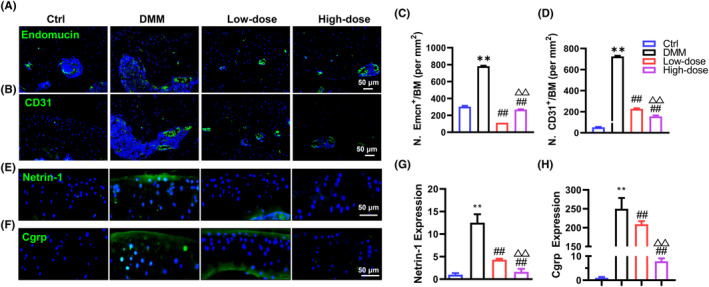
α‐Solanine impairs angiogenesis and sensory nerve ingrowth in subchondral bone of OA mice. (A–D) IF staining and quantitative results of Emcn‐positive and CD31‐positive cells in subchondral bone marrow at 8 weeks post‐DMM surgery. (E–H) IF staining and quantitative results of Netrin‐1 and Cgrp‐positive cells in the subchondral bone. Data are presented as mean ± SD. Three independent experiments were performed in triplicates. *n* = 5 per group. **p < 0.01, compared with Ctrl mice; ^##^ *p* < 0.01, compared with OA mice; ^△^ *p* < 0.05, ^△△^ *p* < 0.01, compared with mice treated with low‐dose α‐Solanine (0.5 μM).

Aberrant angiogenesis and innervation in subchondral bone are key steps in subchondral bone remodelling in OA.^17^, ^31^, ^32^ Subchondral bone remodelling has been shown to occur prior to cartilage degeneration, and the infiltration of type H blood vessels expressing CD31 and Emcn has been recognized as a clinical characteristic of OA.^33^ Then, we analysed the impact of α‐solanine on angiogenesis in the subchondral bone of OA mice. IF results using CD31 and Emcn as indicators of type H blood vessels demonstrated that α‐solanine significantly reduced the increased number of CD31^hi^ and Emcn^hi^ blood vessels in the subchondral bone of OA mice (Figure 4A–D).

In addition, emerging evidence suggests that osteoclasts in the subchondral bone contribute to the sprouting of sensory nerves through the secretion of Netrin‐1, thus triggering OA‐associated pain.^17^ In agreement with previous findings,^10^, ^11^ we found that DMM surgery induced Netrin‐1 expression in the subchondral bone marrow of OA mice, and α‐solanine treatment significantly reduced Netrin‐1 expression in a dose‐dependent manner (Figure 4E,G). Similarly, IF analysis‐targeting Cgrp, a classic molecular marker of sensory neurons, confirmed that α‐solanine administration resulted in a significantly lower percentage of Cgrp^+^ sensory neurons in the subchondral bone relative to OA mice (Figure 4F,H). Overall, these data suggest that α‐solanine can effectively prevent aberrant angiogenesis and innervation in the subchondral bone of OA mice, and the high dosage exhibits greater efficacy than the lower dosage.

### 3.5 α‐Solanine inhibits NF‐κB signalling activity

**FIGURE 5:**
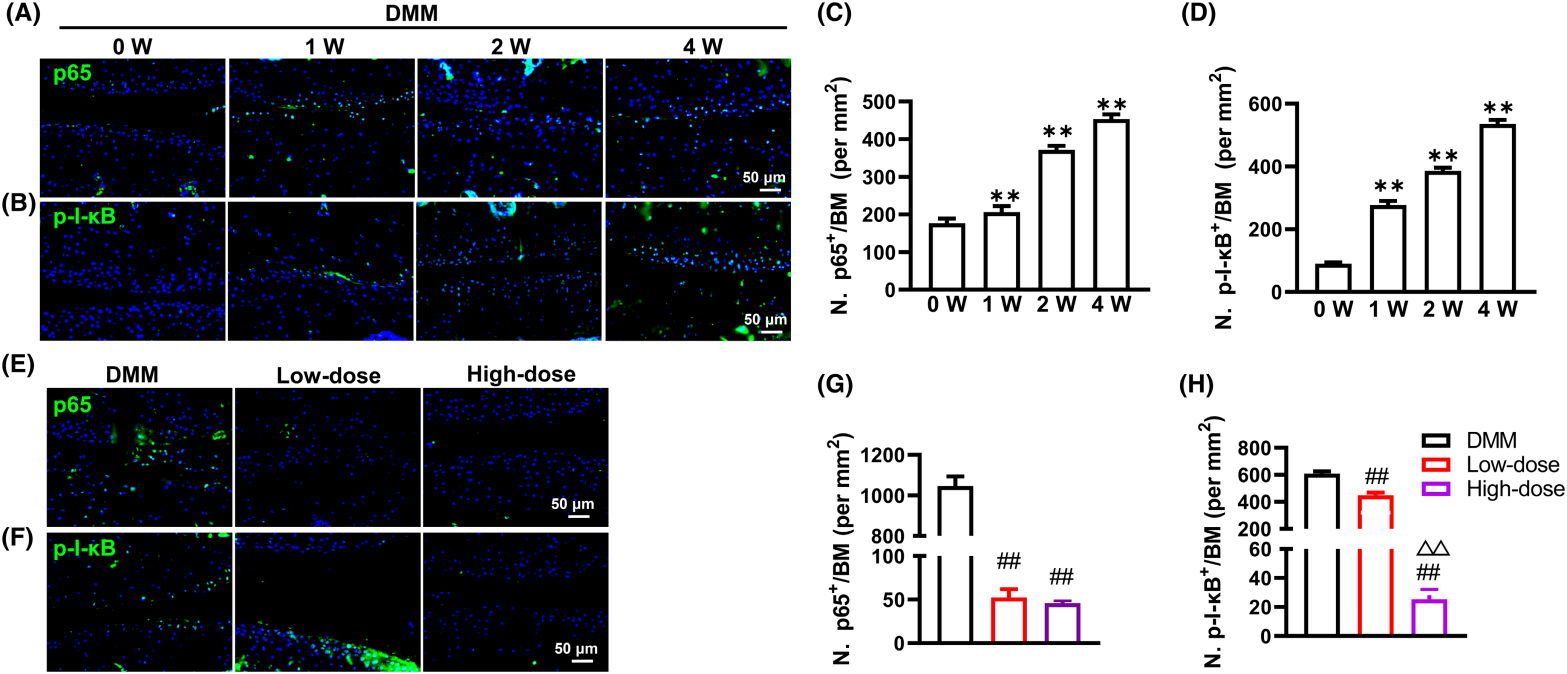
α‐Solanine inhibits DMM‐induced activation of NF‐κB signalling. (A–D) IF staining and quantitative results of p65 and p‐I‐κB in articular cartilage. (E–H) IF staining and quantitative results of p65 and p‐I‐κB in articular cartilage of OA mice 8 weeks after α‐solanine treatment. Data are presented as mean ± SD. Three independent experiments were performed in triplicates. *n* = 5 per group. **p < 0.01, compared with 0‐week mice; ^##^ *p* < 0.01, compared with OA mice; ^△△^ *p* < 0.01, compared with mice receiving low‐dose α‐solanine (0.5 μM).

To further investigate the involvement of NF‐κB signalling in the protective effects of α‐solanine against OA development, we examined the expression of p65 and p‐I‐κB in articular cartilage of OA mice 0–4 weeks after DMM surgery using IF analysis. As expected, we found a progressive increase in the expression of p65 and p‐I‐κB during the early stages of OA modelling, especially at 2–4 weeks (Figure 5A–D). Importantly, α‐solanine treatment potently impeded the nuclear translocation of p65 and reduced p‐I‐κB levels compared to the DMM group, and the high‐dose α‐solanine treatment displayed stronger inhibition of p‐I‐κB compared to the low‐dose α‐solanine treatment (Figure 5E–H). Taken together, these results indicate that α‐solanine treatment could suppress NF‐κB signalling in chondrocytes of OA mice.

## 4 DISCUSSION

**FIGURE 6:**
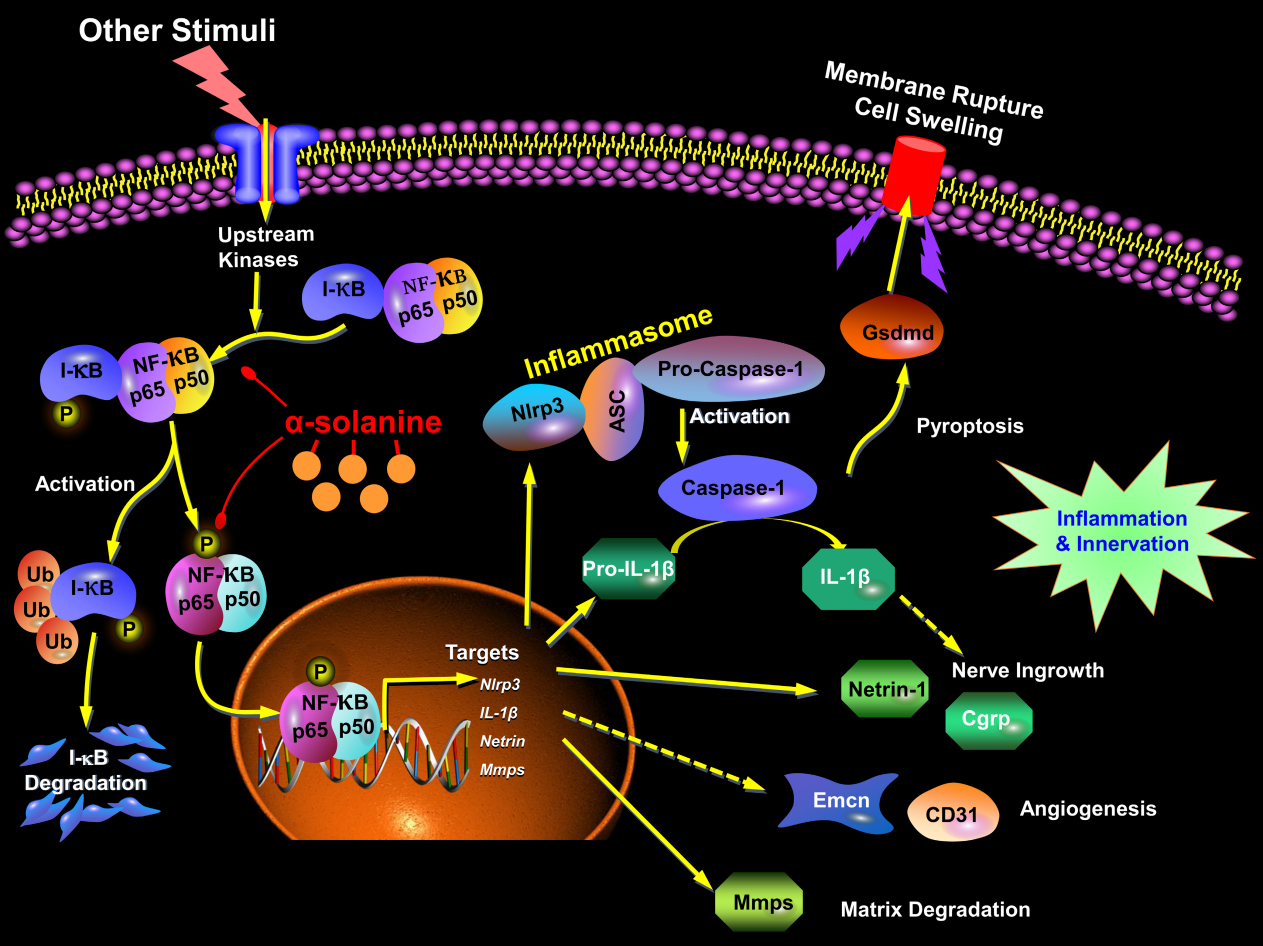
Schematic working model illustrating the α‐solanine‐mediated protection against OA progression by targeting NF‐κB signalling.

OA is a commonly occurring degenerative joint condition characterized by the degeneration of articular cartilage, formation of osteophyte, sclerosis of subchondral bone, angiogenesis and innervation.^5^, ^34^ Current strategies for managing symptomatic OA have limited therapeutic effects and often lead to progressive pathological alterations in the joints, with no recognized disease‐modifying approaches available. In the present study, we present the therapeutic effects of α‐solanine on the progression of OA in mice by inhibiting osteophyte formation, articular cartilage degradation and subchondral bone deterioration. Importantly, α‐solanine mitigated cartilage erosion and proteoglycan loss as well as chondrocyte pyroptosis in articular cartilage. Furthermore, α‐solanine can reduce the number of type H blood vessels and the recruitment of Cgrp^+^ sensory neurons in the subchondral bone. Mechanistic analysis revealed that α‐solanine significantly inactivated the activity of NF‐κB signalling, a crucial upstream stimulator of chondrocyte pyroptosis and dysfunction and sensory nerve innervation in the subchondral bone. To the best of our knowledge, our findings provide novel in vivo evidence supporting that α‐solanine could ameliorate OA development (Figure 6).

Potato is a staple food in China and Western countries.^35^, ^36^ Increasing evidence suggests that potatoes possess health‐enhancing properties, including anti‐inflammatory, antioxidant and anticancer activities.^37^, ^38^ Emerging evidence has revealed that consuming one to two teaspoons of potato juice before each meal can help alleviate arthritis symptoms.^19^ Furthermore, OA patients can relieve arthritis pain and inflammation by drinking four cups of boiled potato peeling water a day.^19^ As one of the main extracts of potatoes, α‐solanine has been reported to exhibit significant anti‐inflammatory and anti‐tumour properties.^39^, ^40^ Growing evidence from in vitro and in vivo experiments suggests that α‐solanine has anti‐inflammatory activity by suppressing pro‐inflammatory mediators induced by LPS.^21^, ^39^ Consistent with these findings,^21^, ^39^ our research demonstrated that α‐solanine can decrease the excessive production of IL‐1β in the articular cartilage of OA mice, and its anti‐IL‐1β effect was dose‐dependent, suggesting that α‐solanine might be beneficial in mitigating the inflammation response during the progression of OA.

Although the pathophysiology of OA is multifactorial and complex, pain remains a key focus in clinical management. Pyroptosis, an inherently inflammatory form of cell death that is morphologically and mechanistically distinct from other forms of cell death, is characterized by caspase‐1‐mediated inflammasome activation and the release of pyrogenic cytokines, particularly IL‐1β.^10^, ^11^ Accumulating evidence from clinical studies on OA patients and animal models suggests that chondrocyte pyroptosis mediated by caspase‐1 regulates the progression of OA, and *Gsdmd* deficiency can reverse this pathogenesis.^41^, ^42^ Our previous studies have shown that inhibiting chondrocyte pyroptosis significantly improves OA progression, suggesting that targeting chondrocyte pyroptosis could be a therapeutic approach for OA.^10^, ^11^ In line with these findings, here we identified that α‐solanine could potently downregulate the levels of caspase‐1 and its downstream effector IL‐1β in articular chondrocytes of OA mice, comparable to the level observed in Ctrl mice, suggesting that α‐solanine may act as an effective caspase‐1 inhibitor to protect against OA progression by targeting caspase‐1‐mediated inflammasome activation.

While cartilage itself is non‐neural, the subchondral bone is considered to be the main source of nociceptive stimulation in OA and is thought to contribute to OA pain.^43^ Moreover, growing discoveries suggest that aberrant subchondral bone remodelling in OA patients promotes sensory nerve innervation, partly through osteoclasts‐mediated secretion of Netrin‐1.^17^, ^44^ In the present study, α‐solanine attenuated the expression of Netrin‐1 and reduced the number of Cgrp^+^ sensory neurons in the subchondral bone of OA mice. This decrease in Netrin‐1 expression may be attributed to the inhibition of osteoclast differentiation and activity by α‐solanine, as it has a definite inhibitory effect on macrophages.^21^ To our knowledge, this is the first evidence that α‐solanine could attenuate the generation of nociceptors during abnormal subchondral bone remodelling in OA progression.

In addition, the increased invasion of blood vessels from the subchondral bone into the cartilage regions plays a role in promoting endochondral ossification in OA, while diminished angiogenesis may contribute to the slowdown of this process. An increasing body of evidence suggests that elevated levels of CD31^hi^Emcn^hi^ vascular congestion are present in the subchondral bone of OA, linking angiogenesis with osteogenesis and facilitating the invasion of cartilage areas to endochondral ossification.^3^, ^45^, ^46^ Conversely, restricting angiogenesis has the potential to slow down OA development.^31^, ^47^ Here, we demonstrate that α‐solanine, particularly at a high dose, could impair angiogenesis in the subchondral bone, as indicated by a reduction in the number of CD31^+^ cells and Emcn^+^ cells, suggesting α‐solanine has the potential to attenuate OA progression by inhibiting pathological angiogenesis.

NF‐κB signalling is widely recognized as critical for maintaining cartilage homeostasis and OA development,^48^, ^49^ which regulates a variety of matrix‐degrading enzymes, inflammatory mediators, cytokines and chemokines.^49^, ^50^ Aberrant activation of NF‐κB activity has been implicated in the increase of chondrocyte pyroptosis during OA development, and it also plays a crucial role in regulating the expression of Netrin‐1, which is involved in nervous system growth.^51^, ^52^, ^53^, ^54^ Additionally, numerous in vitro studies have verified that α‐solanine can inhibit NF‐κB signalling.^28^, ^55^ In support of these findings, here we consolidated that α‐solanine significantly suppressed aberrant NF‐κB signalling, which was activated during the early stages of OA progression. Together, our findings demonstrate that α‐solanine suppresses chondrocyte pyroptosis and sensory innervation, ameliorating the pathological progression of OA by inhibiting NF‐κB signalling, which highlights the potential of α‐solanine as a therapeutic agent for OA treatment.

Although our findings are groundbreaking, it is important to acknowledge several limitations. First, glycoalkaloids such as α‐solanine have limited oral bioavailability and vary greatly across species. For instance, hamsters have been found to have better absorption and slower excretion rates of α‐solanine compared to rats.^56^ The metabolic profiles of α‐solanine in mice remain uncharacterized, requiring further investigations to understand its long‐term cumulative toxic effect on knee function and phenotypic characteristics of normal mice without induced OA. Second, in this study, our study focused on the direct therapeutic effects of α‐solanine through intra‐articular injection therapy, which targets local joints and minimizes systemic adverse reactions.^57^ However, different administration routes, such as gavage, intravenous injection and intraperitoneal injection should be explored to further validate our findings and compare them to the effects of normal oral intake of α‐solanine. Lastly, α‐chaconine and α‐solanine account for 95% of total glycoalkaloids in potatoes, and α‐chaconine is generally slightly more than α‐solanine in potatoes (the ratios of α‐chaconine to α‐solanine ranged from 1.2 to 2.6).^58^ Unexpectedly, our latest finding demonstrated that α‐chaconine intake could exacerbate OA progression.^13^ Therefore, it is uncertain if other components in eggplant potatoes, either alone or in combination with α‐solanine, may have similar or better efficacy in ameliorating OA progression. Detailed analyses are necessary to address this issue. The implementation of all these aspects will unprecedently enhance our understanding of α‐solanine's multiple aspects and its potential benefits for OA patients who consume potatoes.

## 5 CONCLUSION

In conclusion, our study collectively elucidates the capacity of α‐solanine to mitigate the progression of OA by weakening the degeneration of articular cartilage and the exacerbation of subchondral bone. Noteworthy, α‐solanine exerts its effects by suppressing chondrocyte pyroptosis and sensory innervation through the inhibition of NF‐κB signalling. Our work offers novel evidence and profound insights into the prospective therapeutic implications of α‐solanine in the context of OA pathogenesis.

## AUTHOR CONTRIBUTIONS

**Jinyi Zhou:** Data curation (equal); formal analysis (equal); investigation (equal); validation (equal). **Jinting Wu:** Data curation (equal); formal analysis (equal); investigation (equal); validation (equal). **Fangda Fu:** Data curation (equal); formal analysis (equal); investigation (equal); validation (equal). **Sai Yao:** Data curation (equal); formal analysis (equal); investigation (equal). **Wenbiao Zheng:** Data curation (equal); resources (equal); software (equal); writing – review and editing (equal). **Weibin Du:** Conceptualization (equal); methodology (equal); writing – review and editing (equal). **Huan Luo:** Conceptualization (equal); methodology (equal); resources (equal); writing – review and editing (equal). **Hongting Jin:** Data curation (equal); software (equal); writing – review and editing (equal). **Peijian Tong:** Data curation (equal); software (equal); writing – review and editing (equal). **Chengliang Wu:** Data curation (equal); software (equal); writing – review and editing (equal). **Hongfeng Ruan:** Conceptualization (equal); methodology (equal); resources (equal); writing – review and editing (equal).

## FUNDING INFORMATION

This study was financially supported by Natural Science Foundation of Zhejiang Province (No.: LY22H270003 and LQ23H270003), Joint Funds of the Zhejiang Provincial Natural Science Foundation of China under Grant No. BY24H290014, No. LBY22H270008 and LBY21H060002, Traditional Chinese Medical Administration of Zhejiang Province (No.: 2023ZR019, 2022ZX005, 2022ZB119 and 2021ZB090), Zhejiang Medical and Health Science and Technology Project (No.: 2023RC194, 2023KY235 and 2021KY222), Zhejiang Pharmaceutical Association Hospital Pharmacy Special Project (No. 2023ZYY11), Research Project of Zhejiang Chinese Medical University (No.: 2023JKZKTS27, 2023JKZKTS40 and 2021JKZDZC02), Research Project of Zhejiang Chinese Medical University Affiliated Hospital (No.: 2023FSYYZZ02, 2023FSYYZY40, 2022FSYYZZ05 and 2022FSYYZQ02).

## CONFLICT OF INTEREST STATEMENT

The authors confirm that there are no conflicts of interest.

## Data Availability

The original data supporting the conclusions of this article will be provided by the authors, without undue reservation.
